# Superhydrophobic Polypropylene Functionalized with Nanoparticles for Efficient Fast Static and Dynamic Separation of Spilled Oil from Water

**DOI:** 10.1002/gch2.201800115

**Published:** 2019-04-26

**Authors:** Nadeem Baig, Tawfik A. Saleh

**Affiliations:** ^1^ Chemistry Department King Fahd University of Petroleum & Minerals Dhahran 31261 Saudi Arabia

**Keywords:** dynamic separation, fibers, hazardous materials, pollutants, superhydrophobic, superoleophilic

## Abstract

Frequent oil spills not only threaten the ecosystem, but they are also a waste of a valuable source of energy. There is an urgent need to develop materials that can readily remove spilled oil from water bodies and also have the capacity to collect it for energy applications. Herein, a superhydrophobic fiber of functionalized polypropylene is engineered with the help of palmitic acid interaction with incorporated copper oxide nanoparticles. The successful development of functionalized polypropylene is confirmed by Fourier‐transform infrared spectroscopy, X‐ray photoelectron spectroscopy, and energy‐dispersive X‐ray spectroscopy. The scanning electron microscopy images reveal that the surface roughness of the polypropylene is enhanced after functionalization. The optimized functionalized polypropylene displays an ultrahydrophobic surface with a water contact angle of 162.42°. The functionalized polyprolyene displays good absorption capacity. It has the capacity to take 30 to 40 times its own weight in oils and nonpolar organic solvents, which makes it useful for small spills. With a flux of 11 204 Lm^−2^ h^−1^, functionalized polypropylene is as an ideal material for the dynamic separation of oil spills from water. It also has excellent selectivity towards oil, water rejection, and oil absorption capacity.

## 1 Introduction

In recent environmental challenges, water pollution has become a major challenge due to rapid industrialization and urbanization.^1^ The oil films on the surface of the water may affect aquatic systems by decreasing the dissolved oxygen and obstructing the rehydration process. Recent oil spill incidents such as Exxon Valdez, Deepwater Horizon, IXTOC 1, and the first Gulf War has proved catastrophic for the marine echosystem.^2^, ^3^, ^4^ Apart from this, the spilled oil and organic contaminants may result in fire and explosions due to their high flammability which may bring destructive results.^5^ The conventional techniques used for the separation of oil/water mixtures are not limited to flotation, gravity‐based separation, and skimming.^6^ Oil burning has also been conventionally used to eliminate oil spilled contents from water. These conventional methodologies have their own limitations including low efficiency, high‐cost operation, and the potential to cause the addition of secondary pollutants.^7^ Specifically, the burning of oil is responsible for the addition of secondary environmental pollutants and also the destruction of valuable oil. Smart materials with a proper design are desperately required to effectively separate organic contamination and oil spills from water.

Oil/water separation is an interfacial phenomenon, and as a result, the material with special wettability has attracted great attention from researchers.^8^ Superwettability plays a crucial role in the separation of oil and water. Superwettable surfaces are recognized as either superhydrophobic or superhydrophilic. Surfaces with superhydrophobic or superhydrophilic characteristics can selectively allow the passage of oil and water, respectively. The wettability of solid surfaces can be controlled by the rational design of structural geometry and chemical composition. Recently, significant progress has been made in the development of wettable surfaces. For the generation of superwettable surfaces two criteria need to be met: (a) Generating a micro‐ and nanostructured surface roughness; (b) Matrix free energy regulation at a suitable threshold value.^9^ Surface roughness is a crucial feature in the specific wettability of solid materials.^10^ The construction of nanostructures on the solid surface plays an important role in imparting superhydrophobic behavior to the surface.^11^

The surfaces of various support materials^12^ such as foam/sponges,^13^, ^14^ meshes,^15^ cotton,^16^ nonwoven fabrics,^17^ and woven fabrics^18^ are being exploited to develop superhydrophobic surfaces for effective oil and water separation. The range of material used to develop hydrophobic surfaces includes graphene,^19^ carbon nanofiber,^20^, ^21^ nanodiamonds,^22^ metal nanoparticles,^23^ metal oxide nanoparticles,^24^ silica nanoparticles,^25^ and polymers.^26^ Hydrophobic surfaces can be generated by using the hydrothermal method,^27^ the chemical vapor deposition method,^28^ layer by layer assembly,^29^ spraying,^8^, ^16^ and dip and drop coating methods.^30^, ^31^ Polypropylene is a nonpolar polymer and has also received importance in the field of oil and water separation. Wang and Uyama developed hydrophobic macroporous polypropylene sponges which displayed a water contact angle of 130°.^32^

In this work, the polypropylene fibers were functionalized to achieve the superhydrophobic surfaces. The superhydrophobic surface was attained in a two‐step process. In the first step, the copper oxide nanomaterials were incorporated into the polypropylene fibers. In the second step, the polypropylene fibers through the incorporated nanomaterials were functionalized with palmitic acid. The nonfunctionalized polypropylene fibers hydrophobicity was not satisfactory. After functionalization, the polypropylene fibers displayed superhydrophobic behavior and a substantially high water contact angle of 162.42°. The surfaces of the functionalized polypropylene took the oil very fast as it touched the surface.

## 2 Results and Discussion

### 2.1 Surface Analysis of the Nonfunctionalized and the Functionalized Polypropylene

**Figure 1:**
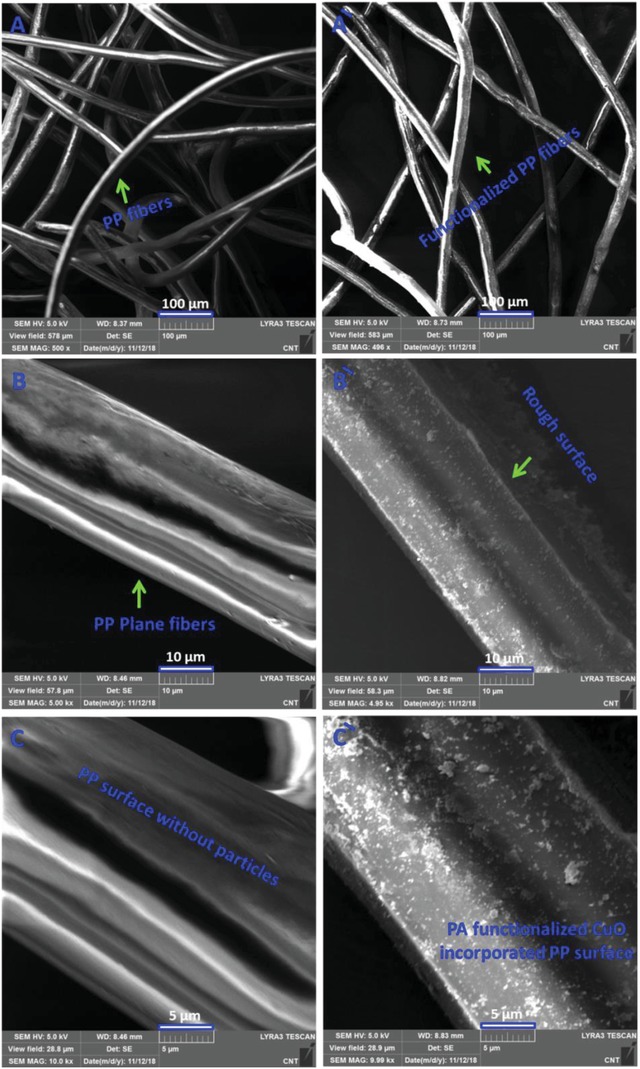
SEM images at different magnification of A–C) PP and Á–Ć) PACuOPP.

Scanning electron microscopy (SEM) was used for the morphological analysis of functionalized and nonfunctionalized polypropylene. The polypropylene fibers appeared somehow planner without any roughness in the absence of functionalization. There were no micrometer or nanometer scale structures observed on the surface of the nonfunctionalized polypropylene. In the case of functionalized polypropylene, a micro/nanoscale structure was detected on the surface of the fibers (**Figure** **1**). The presence of micro and nanoscale materials actually incorporated copper oxide particles which are functionalized with the palmitic acid to enhance the hydrophobicity of the material.

**Figure 2:**
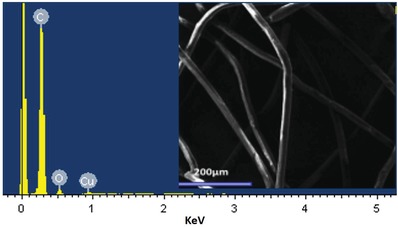
EDX spectra of the PACuO_500_PP.

**Figure 3:**
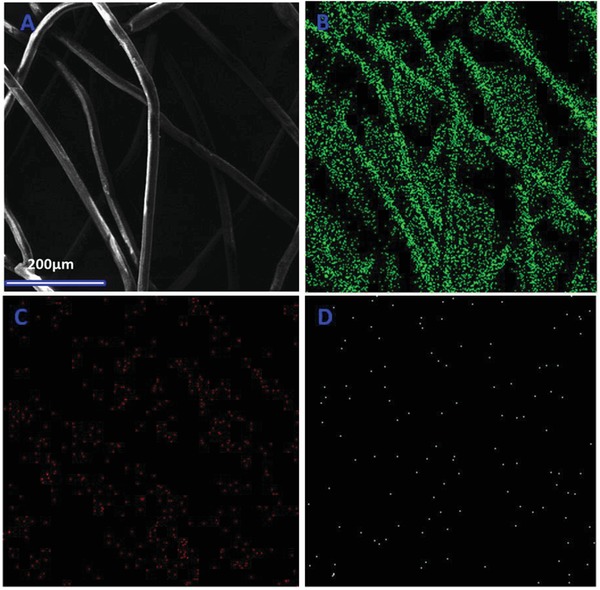
Mapping of the PACuO_500_PP. A) Electron image of the mapping under observation. B) C, C) O, and D) Cu.

The successful incorporation of the copper oxide on the surface of the polypropylene was furthermore investigated by using energy‐dispersive X‐ray spectroscopy (EDX) analysis. The EDX spectrum confirmed the presence of the copper and oxygen apart from carbon in the functionalized polypropylene fibers (**Figure** **2**). The effective incorporation of the copper oxide on the polypropylene is a crucial point to obtain highly functionalized polypropylene. The copper oxide is directly involved in the functionalization of palmitic acid on the surface of the polypropylene fibers. Due to this reason, the incorporation of the copper oxide throughout the fibers of the polypropylene is a mandatory condition to obtain superhydrophobic surfaces. To ensure the uniform distribution of the copper oxide, the EDX mapping was recorded which provided the information about the distribution of the C, Cu, and O in the PACuOPP. As for the polypropylene and palmitic acid, most of it consists of carbon atoms and EDX mapping has demonstrated the intensive presence of carbon atoms (**Figure** **3**B). The EDX mapping of the PACuOPP also revealed a uniform distribution of the copper atoms which follows the shape of the polypropylene fibers (Figure 3D). The mapping revealed that the copper is uniformly incorporated on the surface of the polypropylene.

**Figure 4:**
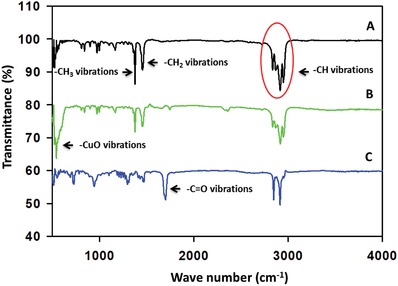
FTIR spectra of A) PP, B) CuO‐PP, and C) PA‐CuO‐PP.

The polypropylene and its various nanomaterial functionalized surfaces were investigated by using Fourier‐transform infrared spectroscopy (FTIR). The FTIR spectra of the polypropylene displayed its characteristics peak. The stretching and the rocking vibrations of the –CH_2_ and –CH_3_ appeared at 808 and 840 cm^−1^. The vibration peaks at 972 and 997 cm^−1^ appeared due to the rocking and bending of the –CH_3_. The sharp bands at 1376 and 1454 cm^−1^ might have appeared due to the bending vibration of –CH, –CH_2_, and the –CH_3_. The peak which appeared in the range of 2760–3000 cm^−1^ was assigned to the –CH stretching vibration of the polypropylene chain.^33^ In the FTIR spectra of copper oxide nanoparticle incorporated polypropylene, a sharp band appeared at 532 cm^−1^ which was assigned to the Cu–O vibrations in the infrared region (**Figure** **4**B). The FTIR spectra of the PACuOPP have also confirmed the successful functionalization of the polypropylene with the palmitic acid. A sharp carbonyl peak appeared at 1699 cm^−1^ in the PACuOPP which was appeared due to the palmitic acid interaction with the copper oxide incorporated polypropylene (Figure 4D).

**Figure 5:**
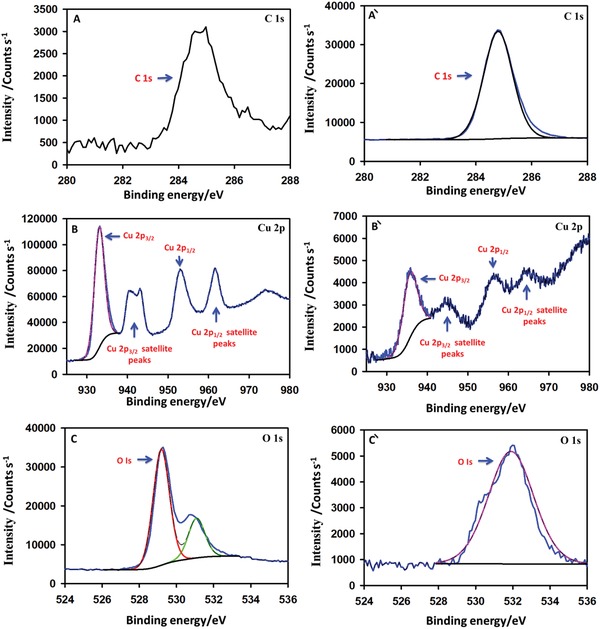
XPS spectra of A–C) CuO nanoparticles, Á–Ć) PA‐CuO_500_‐PP.

The X‐ray photoelectron spectroscopy (XPS) analysis of the CuO nanoparticles and the functionalized polypropylene was scrutinized. The XPS analysis assists in investigating the changes in the binding energy of the copper oxide nanoparticles after incorporation of the polypropylene and functionalization with the palmitic acid. The XPS spectra of the CuO nanoparticles showed an unexpected carbon peak at 284.8 eV which might have appeared due to the carbon impurities from the surroundings (**Figure** **5**A). In the case of PACuO the intensity of the carbon peak was substantially enhanced. This was due to the presence of carbon in the polypropylene and the palmitic acid (Figure 5Á). The Cu2P_3/2_ and Cu 2p_1/2_ peak of the pure copper oxide nanoparticles appeared at 933.05 and 953.16 eV, respectively. The copper oxide characteristic satellite peaks also appeared in the XPS spectra of the copper oxide (Figure 5B). The fitting of the O1s XPS spectra revealed two binding energies at 529.18 and the 531.08 eV. However, in the ase of PACuOPP, the binding energy of the O 1s appeared at 531.9 eV which revealed the oxygen with a different chemical environment in the PACuOPP (Figure 5C). The Cu2P_3/2_ and the Cu 2P_1/2_ peaks binding energy was shifted after interaction of the palmitic acid with the incorporated copper oxide nanoparticles on the polypropylene fibers. The binding energy of the Cu2P_3/2_ and the Cu2P_1/2_ was shifted from 933.05 and 953.16 eV to 935.68 and 956.15 eV. After fitting for the Cu2P_3/2_ in both cases a single peak appeared that might reveal the presence of copper with the same oxidation state. The XPS study with the informative binding energy shifts has shown the successful incorporation of the CuO nanoparticles on the polypropylene and the possible interaction of the palmitic acid with the copper oxide incorporated polypropylene.

### 2.2 Evaluation of Surface Hydrophobicity

**Figure 6:**
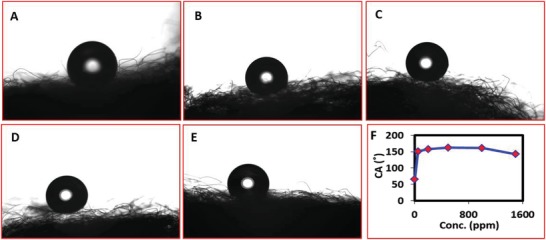
The contact angle of the various PP nanomaterial functionalized: A) PA‐CuO_50_‐PP, B) PA‐CuO_200_‐PP, C) PA‐CuO_500_‐PP, D) PA‐CuO_1000_‐PP, E) PA‐CuO_1500_‐PP. F) Graphical representation of contact angles.

The specific surface wettability is the key factor in the efficient separation of oil from water. Superhydrophobic surfaces have displayed great attraction for oil, as well as for nonpolar organic solvents, and at the same time strongly repel or prevent the water from passing through it. The superhydrophobic surfaces are generally defined with a water contact angle of greater than 150°. Incorporation of the copper oxide on the polypropylene fibers from various concentrations of the finely dispersed copper oxide nanoparticles in ethanol medium has revealed a significant effect in the determination of the hydrophobicity of the functionalized polypropylene. The PP fibers were incorporated with various concentrations of the copper oxide nanoparticles by sonication in 50, 200, 500, 1000, and 1500 ppm solutions of copper oxide. After incorporation of the copper oxide nanoparticles under the same set of conditions, the copper oxide nanoparticle incorporated polypropylenes were functionalized with the palmitic acid. The contact angle of the PP was substantially improved from 64.66° to 150.91° after the functionalization of the CuO_50_PP with the palmitic acid. The contact angle was improved until the CuO incorporation concentration reached 500 PPM. The surface of the PACuO_500_PP and the PACuO_1000_PP displayed an ultra superhydrophobic behavior and displayed a maximum water contact angle of 162.42° and 161.33°, respectively (**Figure** **6**). A lower water contact angle of 142.38° was observed on the surface of the PACuO_1500_PP which might be due to the excessive presence of copper oxide nanoparticles on the polypropylene fiber which slightly reduces the hydrophobic behavior of the surface. The copper oxide presence directly affects the hydrophobicity of the polypropylene as they interact with the palmitic acid. The palmitic acid is the main entity which produced hydrophobic branches on the polypropylene surfaces. For this reason, the copper oxide nanoparticles have a significant effect on the hydrophobicity of the polypropylene. The PACuO_500_PP was selected as the optimum functionalized polypropylene fiber for further investigation of its efficiency for oil and water separation. In case of dynamic water contact angle, it is more fit to the Cassie Baxter model. The water drops on the optimum functionalized surface were difficult to stay on the surface and freely moved on the surface due to the superhydrophobic nature of the functionalized polypropylene. It went off the surface with small tilt and as a result, the functionalized surface has a self‐cleaning capability.

### 2.3 Mechanism Behind Super Surface Hydrophobicity

**Scheme 1:**
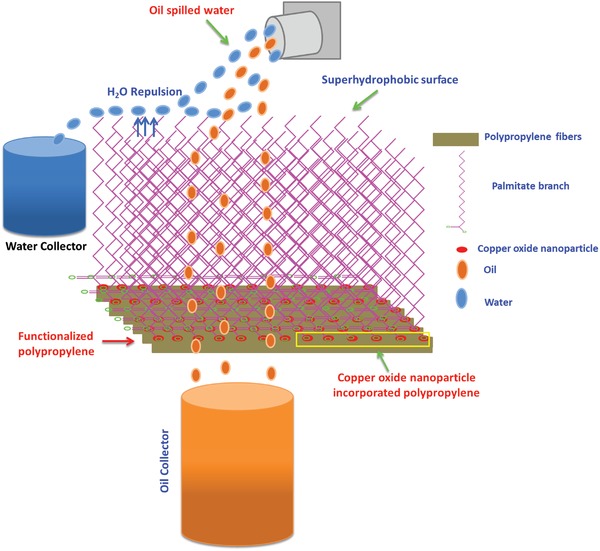
Schematic representation of the oil and water separation mechanism.

Functionalized polypropylene displayed an ultrahydrophobic surface and proved very effective for oil and water separation. The nonfunctionalized polypropylene displayed poor hydrophobic behavior. It was observed that the water drop stuck to the surface and passed through it easily. On the other hand, the functionalized polypropylene strongly repelled the water and readily took the oil. The oil quickly spread over the surface and, during the separation process, the oil rapidly passed through. The ultrahydrophobic behavior of the polypropylene fibers can be explained from the long hydrophobic branches that are generated after interaction with the palmitic acid (C_16_H_32_O_2_). These functionalized polypropylenes work on the principle of “like physically interacted with like.” On this principle, the nonpolar organic solvents and the oil rapidly pass through these superhydrophobic branches of the polypropylene. However, the water is polar in nature and was completely rejected by the low energy nonpolar surface of the functionalized polypropylene. The complete mechanism for the separation of oil and water is explained in **Scheme** **1**.

### 2.4 Absorption Capacity and Reusability

**Figure 7:**
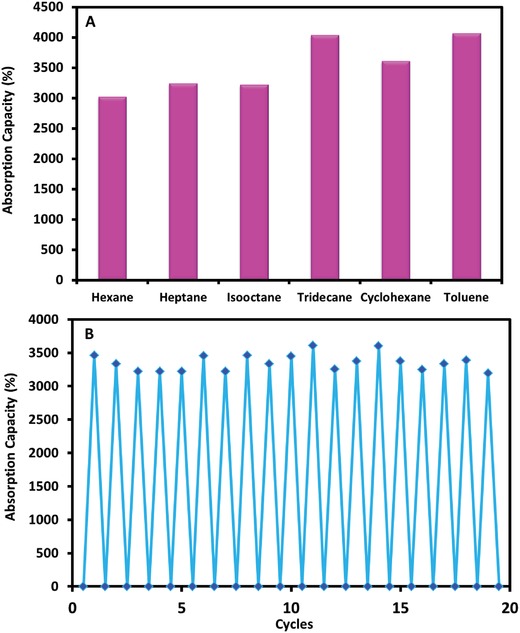
A) Absorption capacity of the PACuO_500_PP for the various nonpolar organic solvents. B) Reusability analysis of the PA‐CuO_500_‐PP for the heptane.

The palmitic acid functionalized nanomaterial incorporated polypropylene nanofibers are lightweight and oil rapidly spread into the fibrous network. The polypropylene itself did not display great superhydrophobic behavior; however, after functionalization, the linked branches of the palmitic acid through the incorporated nanomaterial made it selective towards oil. The developed PACuO_500_PP also displayed good absorption capacity for various nonpolar organic solvents such as hexane, heptane, isooctane, tridecane, cyclohexane, and toluene (**Figure** **7**A). The absorption capacity was observed by dipping the functionalized polypropylene into the oil and immediately transferring it into another container to weigh the oil or the organic components. However, the holding capability of the material is not strong enough and oil continuously comes out of the material. However, it has taken enough quantity with it while transferring to the collector container. The percent weight gain ratio (%*R*) for the absorption of various solvents was found to be in the range of 3000–4100%. The percent weight gain ratio was calculated by using the following Equation (1)

(1) $$\%R=\left(W_{1}-W_{0}\right)/W_{0}$$

Where %*R* describes the percent weight gain ratio, *W*_0_ represents the original weight of the functionalized polypropylene before absorption and *W*_1_ represents the weight of the functionalized polypropylene after oil absorption.

The material regeneration and the retaking capability were evaluated several times by dipping into heptane. The functionalized polypropylene was reused continuously 20 times to absorb and release heptane. A small variation in its absorption capacity was observed after multiple uses (Figure 7B). An RSD of 3.75% (*n* = 20) was observed during multiple cycles. The functionalized polypropylene has displayed a good shelf life. The hydrophobicity of the material was kept under observation for a couple of weeks. The hydrophobicity of the material was evaluated by dropping the water on the surface. During each trial, the water drops did not stay on the surface and freely moved down from the surface. This is an indication that material has a good shelf life and can be stored for a longer time.

### 2.5 Dynamic Separation of Spilled Oil from Water

**Figure 8:**
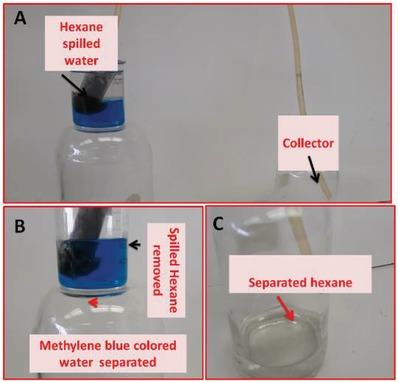
The dynamic separation of the heptane‐spilled, methylene‐blue‐colored. A) Setup of separation B) Methylene‐blue‐colored water after separation. C) Separated hexane.

Oil spill incidents not only pollute water bodies but, at the same time, they also waste a valuable energy source. It is extremely important to recollect the spilled oil and make it reusable in the energy sector. The material which collects the oil without damaging the oil not only protects marine life from its direct effects but also reduces the chances of the addition of secondary pollutants into the environment. Moreover, the nondestructive method also provides a highly desired opportunity to recollect the spilled oil for energy applications. The functionalized polypropylene fibers appeared to be soft in nature. Due to the flexibility of the functionalized propylene, it provides an opportunity to be fixed into any tube and form a dynamic system for the continuous removal of oil spills from the water. An artificial hexane spill in methylene blue colored water was prepared to evaluate the capability of functionalized polypropylene for the dynamic removal of oil from water. A peristaltic pump was used to remove the spilled oil from water. The functionalized material was more than half dipped into the water and the rest of it was kept in hexane to provide the opportunity for the water and hexane to pass simultaneously or selectively from the functionalized polypropylene (**Figure** **8**). The hexane moves very fast from the fluffy functionalized polypropylene while water was not allowed to pass through it. The fast passage of the hexane through it was due to the superoleophilic nature of the functionalized polypropylene fibers. After the removal of the hexane completely from the water, the functionalized polypropylene still did not allow the water to pass. The fluffy superhydrophobic nature of the functionalized polypropylene facilitates the fast passage of the oil. The separated oil can be collected in the collector container for reuse (Figure 8D).

### 2.6 High Flux and Absorption Capacity of the Functionalized Polypropylene

The functionalized polypropylene has carried out many features which make it a unique material for the application of oil and water separation. The superhydrophobic nature of functionalized polypropylene provides for the rapid and fast passage of the oil through it and prevents the passage of water through it during dynamic separation. The fluffy nature of the functionalized polypropylene helps it to fit into the dynamic setup of water and oil separation very easily. The PACuO_500_PP offered a high flux of 11 204 Lm^−2^ h^−1^ during the separation of hexane from the water. This flux was substantially high compared to the previously reported value. Tai et al. developed a membrane that has displayed a contact angle of 144.2° and a hexane flux of 2648.8 ± 89.7 Lm^−2^ h^−1^.^34^ The TPU microfiber membrane and the TPU‐PNIPAM‐3.6 membrane displayed oil fluxes of 4659 and 503 Lm^−2^ h^−1^, respectively.^35^ The functionalized polypropylene also displayed absorption behavior. It can be applied for small spills of oil where dynamic separation is not possible. It displayed a good capacity to take a nonpolar component with it. The absorption capacity was found to be better or comparable with the literature (**Table** **1**). The better performance of the functionalized polypropylene can be explained by its high water contact angle of 162.42° which was much higher than the hydrophobic material described in Table 1.

**Table 1:** Comparison of the functionalized polypropylene with the previously reported literature

SN.	Hydrophobic material	Preparation methodology	Absorption capacity (Hexane)	Water contact angle	Ref.
1	MTMS–DMDMS gels	Sol–gel process	6	152.6°	36
2	MnO_2_/p(BA‐*co*‐BMA‐*co*‐MMA) hybrid resins	Combination of the hydrothermal method and microwave polymerization method	2.07	135°	37
4	3D zz‐PS/GR/PU	Polymerization	9	150°	26
5	Swellable porous PDMS/MWNTs	Hard template approach	15.05	153°	38
6	Graphene aerogel	Freeze drying method	25	–	39
7	Carbon Soot sponge	Flame combustion, Dip coating	25	144°	40
8	Al_2_O_3_/PUF foam sponge	Hydrothermal method	7	144°	41
9	CNT/PDMS‐coated PU sponge	Dip coating method	15	140°	42
10	PACuO_500_PP	Sonicated and a heat‐assisted dip coating method	30	162.42°	This work

**Scheme 2:**
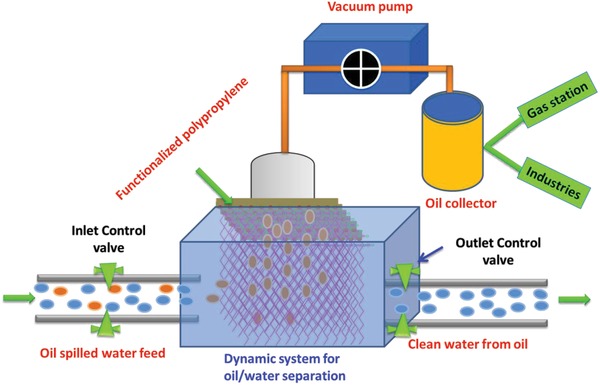
An illustration of the dynamic‐feed‐based model for separation of a large volume of spilled oil from water.

This material can be used to build a commercial setup in which oil containing feed enters from one side into a cleaner unit where it will pass through the PACuO_500_PP and the feed valve will be kept close for some time. During this time, the functionalized polypropylene will take out oil with the help of external pressure and store it in a collector for reuse. After ensuring complete oil removal, the outlet valve will be opened which will allow the passage of clean water (**Scheme** **2**).

## 3 Conclusion

Water pollution is a serious challenge to the healthy sustainability of the ecosystem. Water pollution has direct and indirect lethal effects on marine and human life. Major contributors to water pollution are the oil and the organic spills from industries, households, and unwanted incidents which happen during the offshore movement of oil from one place to another. In recent years some serious efforts have continued to develop materials that can cope with this sort of pollution efficiently and cost‐effectively. In this work, an efficient superhydrophobic polypropylene was developed which displayed an excellent capacity to deal with minor and major oil spills incidents. The copper oxide nanoparticles were incorporated on the polypropylene surface of the fibers which were later on functionalized with palmitic acid to attain the superhydrophobic surface. The optimized functionalized polypropylene demonstrated a very high water contact angle of 162.42°. The functionalized polypropylene was thoroughly investigated by FTIR, XPS, EDX, and SEM. During the dynamic separation of the oil/water mixture, it allowed the fast passage of the oil while it completely prevented the water from passing through it. It offered a high flux of 11 204 Lm^−2^ h^−1^ during the separation of the hexane/water. The functionalized polypropylene also displayed good absorption capability of 30 to 40 times its own weight. The flexibility and absorption capacity of functionalized polypropylene, along with the dynamic separation with the high flux, make it a valuable material for oil and water separation.

## 4 Materials and Methods

*Materials*: Palmitic acid was purchased from Riedel‐de Haën (Germany). Copper oxide was acquired from EMFUTUR. Toluene (LiChrosolv) and Ethanol were obtained from Merck (Germany). Isooctane and tridecane were purchased from Fluka. Hexane, heptane, and cyclohexane were obtained from Sigma‐Aldrich (Germany).

*Instrumentation*: Sonication during the synthesis process was done with a Derui Ultrasonic cleaner. The weight of the various materials was measured by using a Mettler AE 200 weighing balance. A Blue M oven was used for heating purposes. The SEM images of the various materials were recorded by using a TESCAN LYRA 3 field emission scanning electron microscope. A Thermo Scientific Nicolet iS10 instrument was used to collect the IR information of the base materials and the prepared samples. The water contact angle on the various surfaces was recorded and measured by using the OneAttension Tensiometer from Biolin Scientific.

*Development of Functionalized Polypropylene*: Polypropylene was functionalized in two steps. In the first step, copper oxide nanoparticles were incorporated into the polypropylene. Various concentrations of copper oxide nanomaterial; 50, 200, 500, 1000, and 1500 ppm was prepared and sonicated for 30 min to get a fine dispersion of the nanomaterial. The ethanol was used as a medium for the fine dispersion of nanoparticles with the help of the sonication. The polypropylene fibers were dipped into the various concentrations of the copper oxide nanoparticles and continuously sonicated for half an hour. During the sonication process, the copper oxide nanoparticles were uniformly distributed on the polypropylene fibers. After incorporation of the copper oxide nanoparticles, the dispersion liquid was removed and heated at 60 °C in the oven. It was further heated at 80 °C to improve the stability of the copper oxide nanoparticles on the surface of the polypropylene fibers. The 0.1 m solution of palmitic acid was prepared in the ethanol. The copper oxide incorporated polypropylene was dipped into 0.1 m palmitic acid solution. After 10 min, the functionalized polypropylene was taken out and washed extensively with ethanol to remove the excessive and nonfunctionalized palmitic acid from the copper oxide incorporated polypropylene surface. It was dried and heated in the oven to attain a superhydrophobic functionalized polypropylene for the separation of the oil and water.

## Conflict of Interest

The authors declare no conflict of interest.
